# Reviews and Bibliographical Notices

**Published:** 1882-01

**Authors:** 


### REVIEWS AND BIBLIOGRAPHICAL NOTICES.

THE NURSE AND MOTHER; *a manual for the guidance of monthly nurses and mothers*, by Walter Coles, M. D. Published by J. H. Chambers & Co., St. Louis.

In the brief manual before us, comprising but 153 pages, the author has endeavored to impart some useful information, to correct some barbarous superstitions, and to place in just and proper relations the duties and responsibilities belonging to monthly nurses, mothers and physicians. How well he has succeded it will be for the reader to judge. There are, perhaps, few occasions in medical practice attended by as grave responsibilties as are encountered in the lying-in chamber; and the nonchalance with which every old granny assumes the office of the qualified nurse or skilled *accoucheur*, not much less than the tacit consent with which the more than ordinarily intelligent of our community resign the lives of their dear ones, is appalling. Again, the perfect confidence which many of our ladies repose in Mrs.———, a splendid nurse, &c., without any other qualification for the duties than that of being *fashionable*, is equally deplorable. The experience of every physician abounds with instances in which the culpable ignorance, gross superstition or great carelessness on the part of the nurse, has jeopardized the life of mother and child: or in which some incautious remark has produced very unnecessary alarm, or perhaps, indeed, the nurse magnifies her own importance to the extent either of countermanding the directions of the medical attendant, or of criticising them, to the great detriment of the physician. The remedy for these evils is, undoubtedly, to educate both the people and the nurses, and just in proportion to the coolness and intelligence of the nurse will her usefulness be found to increase. Dr. Coles has thoroughly appreciated this state of affairs, and points out clearly the reciprocal relations between nurse, patient and physician. A chapter is devoted to preparations for labor, another to labor itself, to the management of the lying-in woman, accidents and emergencies, and finally one to the management of the child. Every nurse and mother will find food for reflection in the pages of this little book, which is an honest effort to correct many of the abuses to which the puerperal woman is liable. Its style is clear, plain and popular, and will be appreciated by nurses and mothers who are anxious and willing to learn.

*The Prophylactic and Therapeutic Value of Quinine in Gynecic and Obstetric Practice.* By Henry F. Campbell, M. D., Augusta, Ga. Reprint from *Gynæcological Transactions*, 1881, pp. 45.

This *brochure* gives the results of the learned author's observation and experience for many years, and is a practical contribution towards estimating and defining the value of quinine in the specialty to which he has given much attention. At the end of his paper he sums up his conclusions, which we quote in another place in this number of the INDEPENDENT PRACTITIONER.

*Registration and Sanitation: Their Value.* A Preliminary Report read before the Board of Health of the State of Georgia. By Henry F. Campbell, M. D., of Augusta, Ga. Republished from the Annual Report of the Board of Health, for the Instruction of the People, pp. 23. A strong argument for the adoption of measures to secure fuller and more accurate registration of disease, and improved methods of sanitation.

*The Atlanta Medical Register* is the name under which the old and well-known *Atlanta Medical and Surgical Journal* now appears. Our veteran friend, Dr. Westmoreland, who has conducted the old *Journal* through many critical periods, has retired from the editorship, and is succeeded by Drs. J. Thad. Johnson and James B. Baird. With the infusion of this new blood, and its change of name, the *Register* will doubtless take on a new growth, and be valued more than ever. We wish it a prosperous new year.

*The Detroit Clinic* is the title of a new claimant for the favor of the profession, which we find on our table. It is to be a weekly and will be published by Geo. S. Davis, who already issues two other journals under his imprint. The managing editor is Dr. H. O. Walker. The first number presents a neat appearance, and we place it on our exchange list with pleasure.

*The Medical News and Abstract*, our excellent contemporary in the sober, quaker dress has been changed into a weekly under the title of *The Medical News.*

*The Wyoming Literary Monthly* is a new aspirant for the favor of the public, in the otherwise unoccupied field of literary study. Number 2 of the magazine for December, 1881, has a well-filled table of contents, the principal articles being studies of The Miracle Plays, Spenser, Walter Scott and Alfred Tennyson. The *Monthly* is a unique publication and deserves success. It is published by C. Wells Moulton, at Buffalo, N. Y. The subscription price is only $2.00 per year.

*Atlantic City as a Winter Health Resort.* By Boardman Reed, M. D., second edition, Philadelphia, 1881, pp. 22.

Contains facts and inferences about the availability of Atlantic City as a winter sanitarium for consumptives, and those suffering from other pulmonary diseases.

*The Sanitary Engineer* is one of the most esteemed of our exchanges. An examination of our pages will show that we frequently borrow some of the good things from its bill of fare to spread before our readers. Beginning four years ago as a sixteen page monthly, it has, after passing through an intermediate stage as a semi-monthly, just made its appearance as a substantial weekly of twenty pages. We wish it the best of success in its endeavor to teach the practical application of correct principles in sanitation.

